# Perspectives on metals-based radioimmunotherapy (RIT): moving forward

**DOI:** 10.7150/thno.57177

**Published:** 2021-04-15

**Authors:** Jordan M. White, Freddy E. Escorcia, Nerissa T. Viola

**Affiliations:** 1 Cancer Biology Graduate Program, Wayne State University School of Medicine, Detroit, MI 48201.; 2 Department of Oncology, Karmanos Cancer Institute, Detroit, MI 48201.; 3 Molecular Imaging Branch, Radiation Oncology Branch, National Cancer Institute, Bethesda, MD 20814.

**Keywords:** radioimmunotherapy, radiopharmaceuticals, targeted radiotherapy, theranostics, oncology, cancer

## Abstract

Radioimmunotherapy (RIT) is FDA-approved for the clinical management of liquid malignancies, however, its use for solid malignancies remains a challenge. The putative benefit of RIT lies in selective targeting of antigens expressed on the tumor surface using monoclonal antibodies, to systemically deliver cytotoxic radionuclides. The past several decades yielded dramatic improvements in the quality, quantity, recent commercial availability of alpha-, beta- and Auger Electron-emitting therapeutic radiometals. Investigators have created new or improved existing bifunctional chelators. These bifunctional chelators bind radiometals and can be coupled to antigen-specific antibodies. In this review, we discuss approaches to develop radiometal-based RITs, including the selection of radiometals, chelators and antibody platforms (i.e. full-length, F(ab')_2_, Fab, minibodies, diabodies, scFv-Fc and nanobodies). We cite examples of the performance of RIT in the clinic, describe challenges to its implementation, and offer insights to address gaps toward translation.

## Introduction

Monoclonal antibodies (mAbs) have been used clinically for therapeutic purposes since the FDA approval of muromonab-CD3 (orthoclone OKT3), an anti-cluster of differentiation 3 (CD3) antibody, in 1986. Over the past 35 years nearly 100 therapeutic mAbs have been approved for either cancer or non-cancer indications with 11 being granted first approval in 2020 1,2. Most antibodies are developed as *naked* therapeutics, with antibody-dependent cellular cytotoxicity (ADCC) and complement activation identified as the two primary mechanisms that drive their efficacy. Despite the development of chimeric and humanized mAbs to mitigate anti-murine antibody (HAMA) response to first generation murine mAbs, tumor response to single agent mAb monotherapy remains underwhelming 3. Thus, alternative strategies have emerged, focusing on increasing therapeutic efficacy and improving clinical benefit for patients by arming mAbs with cytotoxic chemicals or radionuclide warheads 4,5.

Radioimmunotherapy (RIT) has been around for nearly four decades, however, clinical translation has been limited. RITs leverage biomolecule specificity for tumor-specific antigens to deliver therapeutic radionuclides. Full-length mAbs, smaller fragments 6 (i.e. F(ab')_2_, or F(ab)) or new fusion proteins 7 (i.e. scFv, scFv-Fc, minibody, diabody, or nanobodies) are all being developed as targeting scaffolds for RIT. Choosing a specific mAb-based carrier format is critical for optimizing and balancing the therapeutic index (TI), or increasing the absorbed dose in the tumor, while minimizing toxicities in non-target tissues.

Patient selection for RIT is mainly based on the expression of specific tumor antigens that are either predetermined pathologically or via a companion diagnostic. This exemplifies the concept of tailoring precision medicine to the disease: providing the right patient with the right drug at the optimal dose and time. Because hematological malignancies are radiosensitive, and exist in the blood compartment, where the RIT is administered, two RIT agents have been approved for use in B-cell lymphomas. On the other hand, RIT development for solid tumors is fraught with challenges, primarily stemming from poor tumor vascularization, which contributes to heterogeneous delivery and radioresistance. Dose-limiting toxicity of radiosensitive and healthy organs represents an additional challenge to RIT for both solid and liquid tumors 3.

At the time of this writing, there are only two RIT mAbs approved by the FDA for the treatment of relapsed, refractory non-Hodgkin lymphoma: [^90^Y]Y-ibritumomab tiuxetan (Zevalin^®^) and [^131^I]I-tositumomab (Bexxar^®^) approved in 2002 and 2003, respectively 8. Both agents target the CD20 antigen, expressed on B-cells and B-cell malignancies, and deliver β-emitting radionuclides to the disease sites. Zevalin^®^ demonstrated 80% overall response rate (ORR) and 30% complete response rate (CRR) compared to 56% and 16% for the standard of care rituximab (chimeric mAb specific to CD20), respectively 9,10. Bexxar^®^ has shown ORR of 95% and CRR of 75% 11. Despite the clinical approvals and demonstrable benefits of both RITs, use of both waned shortly after approval. Zevalin^®^ use has continued to decrease year-over-year, and Bexxar^®^ was discontinued in 2014 for economic reasons 12. The market failure of both RITs precipitated by challenging logistics, limited referrals, and underlying issues with medical reimbursements in the U.S. The fact that rituximab was an available non-radioactive option for the same indication that fit better with existing clinical workflows further contributed to the lack of use of RIT, despite its superior clinical outcomes 12,13.

Although RIT has been developed using metal and non-metal nuclides, the scope of this review only includes radiometals-based RIT with antibodies or fragments as carriers. Herein, we discuss the preclinical development and design considerations of the RIT agent: 1) the inherent properties of the therapeutic radiometal, 2) bifunctional chelator (BFC) and 3) the antibody platform. Further discussions on preclinical *in vivo* considerations will include assessing toxicity (e.g. maximum tolerated activity (MTA), therapeutic index (TI), organs at risk for radiotoxicity) and strategies to mitigate it (e.g. pretargeting, fractionation). Finally, we will identify and discuss gaps in clinical needs to aid bench scientists to tailor preclinical development of RIT toward clinical translation.

## Therapeutic radiometals

Therapeutic radiometals are selected based on their particle emission, particle range, half-life (t_1/2_), cost, availability, ease of labeling and use 3. Radiometals can have high, intermediate or low linear energy transfer (LET), the amount of energy released by radiation over the path length that the particle is emitted (keV/μm) 14,15. The path length is the distance that the radiation particles can travel, and informs the size of tumors that can be treated within that range of distance 16. Emitted particles are either alpha- (α) particles, beta- (β^-^) particles, or Auger electrons (AE). Table 1 lists inherent physical properties of commonly used therapeutic radiometals in biomedical research.

**Figure 1:**
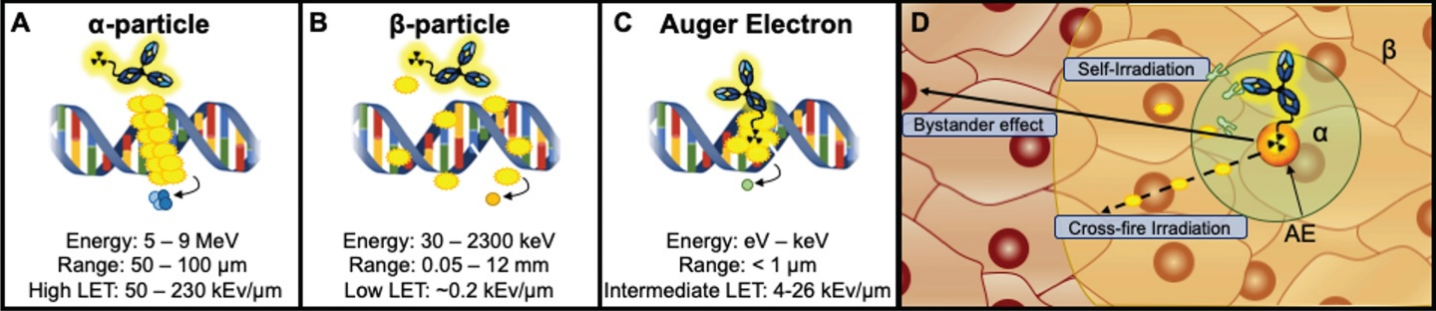
Therapeutic radiometals possess unique decay characteristics resulting in varying energies and ranges in the target tissue referred to as linear energy transfer (LET). **A.** α-particle emitters have the highest LET produced by high MeV level energies and intermediate path lengths (μm). **B.** β-emitters have intermediate energies (keV-MeV) coupled with a long path length (mm) that produce low LET radiation that can traverse ~50 cell diameters. **C.** AE emitters have intermediate LET produced by low energies (1 eV - 1 keV) and distances typically <1μm. **D:** The potential range of the radiation type is depicted in a tumor tissue (β - yellow, α - green, AE - orange). Additionally, radiometal therapeutics have been described to induce toxicity not only in the cell expressing the target antigen (self-irradiation) but also to nontargeted nearby cells by crossfire irradiation. Instances in which cells have not been irradiated but exhibit characteristics similar to irradiated cells are described as a bystander effect.

Alpha-emitting radionuclides release highly energetic ^4^He^2+^ (5-9 MeV) particles that travel short ranges (50-100 μm) 27. They are highly cytotoxic, producing dense clusters of irreparable single or double strand breaks (SSBs/DSBs) in DNA (Figure 1A,D) 16. The combination of high energy and short path length (equivalent to a few cell diameters 27,54) of α-emitting radionuclides (LET 50 - 230 keV/μm) suggest that they may be ideal for targeting smaller solid tumors, such as neoplasms and micrometastases, however α-emitters have also been successfully employed for treatment of larger tumors 14,55. A thorough discussion of the properties of α-emitting radionuclides, their handling and production can be found in a comprehensive review by Poty *et al.* 14.

Beta (β^-^)-particles are intermediate energy electrons that are emitted following a transformation from one proton to a neutron 16. The combination of intermediate energy (30 keV - 2.3 MeV) and longer path length (0.05-12 mm) produce a low LET (~0.2 keV/μm) that can induce a mix of sparse DNA SSBs and DSBs. It is worth noting that the long path length of β^-^-emitters can reach up to ~50 cell diameters 56, which may make them suitable for targeting larger, heterogeneous tumors via cross-fire irradiation, or radiation-induced damage to adjacent nontargeted cells within the range of the RIT (Figure 1B,D) 57.

Finally, AEs are emitted as radioisotopes decay via electron capture. This process occurs following the vacancy of an inner shell electron that is then filled by an electron in an upper shell. A majority of the resulting energy can be released as x-ray energy, however the kinetic energy is transferred to another electron causing the emission from the outer shell 58. AEs have an intermediate LET range (4 - 26 keV/μm) due to the combination of low energy (1 eV - 1 keV) and short path length (< 1 μm) 16. Due to their short range, AEs render optimum DNA damage when localized in or within close proximity to the nuclear compartment of cells (Figure 1C,D) 3. Thus, a mAb carrier with internalizing properties is appropriate. AEs have also been reported to produce “bystander effects”, which cause lethal biological damage to neighboring cells by releasing mediators of cell death 59-61. Figure 1 depicts a schema of different particle emitting RIT agents. A detailed discussion of the biological effects of radionuclides in radioimmunotherapy can be found in reviews by Pouget *et al.* and Ku *et al.* 16,60.

The half-life (t_1/2_) of the radionuclide is critical when designing a radiopharmaceutical. In principle, the physical t_1/2_ of the radiometal should complement the pharmacokinetic t_1/2_ of the mAb or its fragments. The goal is for the carrier molecule to efficiently localize within the tumor for long enough to deposit a concentrated lethal dose of radiation through the complete decay of the radiometal 15. For example, ^212^Pb has a t_1/2_ = 10.6 h and is a β^-^-emitter, but is widely used as an *in situ* generator of its α-emitting daughter isotope ^212^Bi (t_1/2_ = 60.6 m) 14. The t_1/2_ of ^212^Pb is beneficial because it allows for a more facile dose preparation and delivery of over 10-fold greater activity of ^212^Bi 62. To this end, the half-life further impacts logistical requirements such as availability, shipping and radiosynthesis time with only a select number of facilities producing these isotopes 14.

In general, radiometals residualize, or are retained intracellularly within lysosomes or transchelated to intracellular proteins 63. This residualization effect can give certain therapeutic radiometals a unique advantage as they emit DNA-damaging particles within the tumor target 63. However, the non-specific internalization of RITs can also limit efficacy and is problematic for organs that facilitate clearance 64. A study by Tsai *et al.* compared the A11 minibody specific for anti-prostate stem cell antigen (PSCA) labeled with β^-^-emitting ^177^Lutetium (residualizing, t_1/2_ = 6.7 d) vs. ^131^Iodine (non-residualizing, t_1/2_ = 8 d) to determine if radioimmunoconjugate internalization affected the maximum potential activity administered 64. The study specifically utilized the A11-minibody to compare the effects of non-residualizing and residualizing radionuclides because PSCA has previously been shown to have slow internalization kinetics 65. Although both [^177^Lu]Lu-DTPA-A11 and [^131^I]I-A11 exhibited similar cytotoxicity *in vitro*, the *in vivo* analyses showed that [^177^Lu]Lu-DTPA-A11 is a less efficient RIT agent. Dosimetry calculations from the distribution data showed that MTA for [^177^Lu]Lu-DTPA-A11 was established at 7.4 MBq, to offset renal toxicity, but was predicted to be ineffective. In comparison, 37 MBq of [^131^I]I-A11 displayed a tumor response with marginal off-target toxicity.

## Bifunctional chelator

Bifunctional chelators (BFCs) are required to link the mAb and the radiometal. BFCs not only encapsulate the radiometal, but also possess reactive functional groups for covalent binding to the mAb 30. A stable radiometal-BFC complex is paramount for *in vivo* development as it will prevent transchelation of the radiometal with other proteins *in vivo*. Minimizing transchelation is imperative as it prevents nonspecific uptake of the radiometal in off-target organs 66. To establish a thermodynamically stable and kinetically inert radiometal complex, the overall coordination chemistry and size of the metal should be considered 67,68. The nature of the metal, whether it is a soft or hard acid, should be paired with corresponding soft or hard donor atoms from the chelate 68,69.

**Figure 2:**
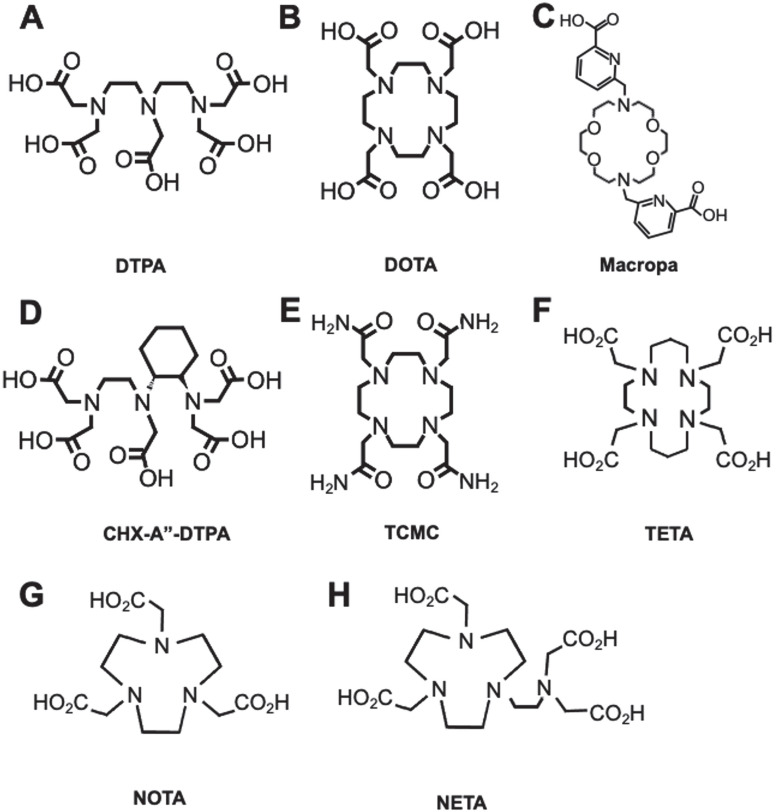
**Common chelators for RIT radioisotopes. A.** DTPA can form an octadentate coordination with three tertiary amine nitrogen donors and five oxygen donors from the carboxylic acid arms. **B.** DOTA chelates metals with four tertiary amine nitrogen donors and four oxygens from carboxylic acid, forming an octacoordinate metal complex. **C.** Macropa is an 18-membered macrocyclic ligand that has shown success for stable chelation of ^225^Ac and ^227^Th. **D.** CHX-A”-DTPA is a derivative of DTPA. **E.** TCMC is a derivative of DOTA with four primary amide pendant arms for stable chelation of ^212^Pb. **F.** TETA is a selective chelator of ^64/67^Cu. **G.** NOTA, and its derivative **H.** NETA is a hexadentate chelator that was utilized for radioisotopes such as ^67^Ga and ^90^Y, respectively.

Current available chelators are either linear or macrocyclic, and radiolabeling strategies are largely temperature and time-dependent, which requires optimization 7. Linear, or acyclic, chelators such as diethylenetriaminepentaacetic acid (DTPA) characteristically have effective complexation efficiencies (Figure 2A) with sufficient radiochemical yields at suitable mAb radiolabeling conditions (e.g. room temperature, < 1 h incubation, physiological pH). Macrocyclic chelators such as 1,4,7,10-tetraazacyclododecane-1,4,7,10-tetraacetic acid (DOTA) are more kinetically stable (Figure 2B), limiting *in vivo* decomplexation of the radiometal and minimizing off-target toxicity. However, macrocycles often need longer time and higher temperatures (> 40 ºC), which can adversely affect the tertiary structure of heat-sensitive mAbs.

A number of chelators have been investigated to encapsulate specific radiometals as noted in Table 1. The general structures of common chelators are illustrated in Figure 2A-H. A review by Price and Orvig highlights appropriate chelate/radiometal matches and discusses each chelate in depth 30. One salient pretargeted RIT (pRIT) study compared DOTA and DTPA labeled with [^177^Lu]LuCl_3_ prior to *in vivo* click conjugation to tumor-bound mAbs 70. pRIT is a multi-step approach in which a mAb targeting a cell surface antigen is administered and allowed to clear over time from nonspecific sites before administration of the radiometal complex that can specifically localize to the antigen-bound mAb 71. The study showed that DOTA is superior to DTPA for chelating [^177^Lu]LuCl_3_, as evidenced by increased tumor uptake (12.0 ± 5.3 %ID/g versus 4.3 ± 1.8 %ID/g at 72 h p.i., respectively). However, a comparison of metal-chelate stability showed that DOTA (93.6 ± 0.9%) was only marginally better than DTPA (85.5 ± 3.0%), after a 48 h incubation, with the latter demonstrating increased retention in kidneys and large intestine. For a thorough discussion on the coordination chemistry and stability of metal-DOTA complexes we direct the readers to a review by Viola-Villegas and Doyle 72.

One of the challenges encountered by metals-based radionuclide therapies, specifically with highly energetic alpha-emitters (e.g. ^225^Ac, ^223^Ra), is the lack of effective and suitable bifunctional chelators that can rapidly complex the metal at suitable temperature conditions and provide long term stability *in vivo*. Despite DOTA remaining the state-of-the-art chelator for most radiometals, complexation conditions are problematic especially when pre-conjugated to the mAb prior to chelation and high heating conditions are required. Recently, Thiele *et al.* demonstrated that an 18-membered macrocyclic ligand (H_2_macropa) (Figure 2C) led to a successful and stable chelation of ^225^Ac after 5 min. incubation at room temperature. In contrast, only 10% of the radiometal was bound to DOTA in these conditions 22. *In vitro* stability was tested by monitoring [^225^Ac]Ac-macropa in the presence of 50-fold excess La^3+^, a lanthanide metal with high affinity for this chelator in human serum. The radiometal complex was found intact over a period of at least 8 days. The study expanded its investigation by labeling ^225^Ac to RPS-070, a prostate specific membrane antigen targeting compound, and trastuzumab, both of which were conjugated to macropa via thiourea linkages. Labeling of the conjugates achieved >99% radiochemical yield after 5 min. Remarkably, the [^225^Ac]Ac-macropa-trastuzumab radioimmunoconjugate remained >99% intact in human serum over 7 d.

The FDA approved [^223^Ra]RaCl_2_ (Xofigo®) in 2013 for palliative treatment for bone metastases in prostate cancer. However, stable chelation has remained difficult. When uncomplexed, it accumulates in areas of high bone turnover and osteoblast activity 73. Abou *et al.* first reported the chelation of ^223^Ra with macropa 18. High radiolabeling efficiencies were achieved at room temperature and with micromolar concentrations of the chelate. Work is currently underway to optimize the stability of the [^223^Ra]Ra-macropa complex when functionalized to peptides and mAbs.

## Antibody-BFC Conjugation Strategies

Various established strategies have been reported and optimized for conjugation of BFC to mAbs via ε-amino groups of lysines. Canonical amine-reactive groups include isothiocyanates (NCS) and activated esters, (e.g. *N*-hydroxysuccinimide esters (NHS-esters)) that produce thiourea and amide linkages, respectively between chelator and mAb. With approximately ~20 solvent accessible lysine residues out of ~80 lysines in a typical IgG scaffold, conjugations tend to be uncontrolled and non-site specific 5,74. Thus, inconsistencies in chelator-to-mAb ratio often produce heterogeneous conjugates, which can dramatically alter the pharmacokinetics of the radioimmunoconjugate 75,76.

Another approach to BFC conjugation utilizes maleimide-functionalized moieties to react with sulfhydryl groups from available cysteines forming thioether linkages. Although still nonspecific, there are four solvent accessible disulfide bonds, which can be reduced with dithiothreitol (DTT) or tris(2-carboxyethyl)phosphine (TCEP), allowing for up to eight linkages per antibody. This strategy provides more moderate control of the conjugation due to the limited number of sites compared to lysine conjugations 75,77. The disadvantage to these thioether linkages is their limited *in vivo* stability 78. Nevertheless, both types of reactions are simple, widely accessible, and have been explored successfully in antibody-drug conjugations.

More recently, unique site-specific approaches were employed for the conjugation of chelators to mAbs 79,80. This approach allows for homogeneous conjugations that result in stoichiometrically uniform attachment of chelators across different batches of conjugations. Importantly, site-specific conjugations abrogate attachment to the antigen binding region of the antibody, preventing loss of immunoreactivity. The four major approaches for site-specific conjugation to antibodies include i) specific amino acids, ii) unnatural amino acids, iii) short peptide tags or iv) glycans 81,82. These strategies have been largely limited to the development of imaging tracers 83-85 or for antibody-drug conjugation 86,87. At the time of writing, only a handful of RIT studies have utilized site-specifically labeled antibodies 79,80,88,89.

### Click chemistry

Click chemistry is a strategy that has been used for the development of pRIT as it provides a rapid labeling approach through bioorthogonal reactions which are inert, or non-reactive, to biological systems. The main goal of pRIT is to prevent off-target toxicities while increasing the TI. Click chemistry reactions that have been examined for pRIT include inverse electron-demand Diels-Alder (IEDDA) cycloaddition, Staudinger ligation and the strain-promoted azide-alkyne cycloaddition 90. The IEDDA cycloaddition reaction widely utilizes tetrazine (Tz) and *trans*-cyclooctene (TCO) pairs where Tz is bound to a specific chelate for the radiometal and the mAbs are bound with TCO 91. It is important to note that attachment of “clickable” functional groups to the mAb is non-specific.

One of the advantages of IEDDA cycloaddition is its utility for *in vivo* pRIT, which typically involves stepwise administration of the reagents. First, the TCO-mAb conjugate is administered to allow sufficient time to localize and accumulate in the tumor and clear from the blood pool and other off-target tissues. This is followed by the administration of the Tz-radionuclide. As the Tz-radionuclide homes in within close proximity to where TCO-mAbs are bound, an *in vivo and in situ* “click” reaction is generated. The pretargeted Tz/TCO click ligation is rapid, with reaction rates of 210-30,000 M^-1^ s^-1^, which allows for rapid clearance of unbound radioligands 92. The concept of administration route, and a more in-depth examination of pRIT, will be further discussed in the preclinical section.

To date, a number of studies have utilized the Tz/TCO strategy for pRIT 31,70,93-96. A study targeting CA19.9 using TCO-conjugated humanized 5B1 compared the use of the α-emitter [^225^Ac]Ac-DOTA-PEG_7_-Tz for pRIT to preconjugated [^225^Ac]Ac-DOTA-PEG_7_-5B1 in pancreatic cancer. No difference in tumor uptake was observed, however, the pRIT approach displayed significantly lower blood and liver concentrations, producing increased tumor-to-spleen and tumor-to-bone radiotracer accumulation ratios 94. In a separate study, LS174T human colon carcinoma xenografts were pretargeted with CC49-TCO, an anti-tumor-associated glycoprotein (TAG-72) antibody, followed by injection of [^212^Pb]Pb-DOTA-Tz 31. Although both the pretargeted CC490-TCO and pre-conjugated [^212^Pb]Pb-TCMC-CC49 exhibited tumor growth inhibition compared to PBS controls, mice administered [^212^Pb]Pb-TCMC-CC49 exhibited faster tumor growth than the pRIT. Additionally, utilization of pRIT allowed for a five-fold increase in activity administered with decreased blood toxicities, which are hypothesized to be a result of decreased circulation time and increased clearance of the smaller [^212^Pb]Pb-DOTA-Tz therapeutic radionuclide.

Rondon *et al.* examined the effects of different polyethylene glycol (PEG) linker lengths as part of the [^177^Lu]Lu-DOTA-Tz construct in a pRIT strategy with a TCO-conjugated anti-carcinoembryonic antigen (CEA) antibody 35A7 targeting peritoneal carcinomatosis of colorectal origin 96. The study first evaluated PEG_n_ linkers with lengths of n = 3, 7, or 11 with the goal of optimizing tumor uptake and clearance profile. [^177^Lu]Lu-DOTA-PEG_3_-Tz exhibited increased clearance in the peritoneum and higher liver accumulation while [^177^Lu]Lu-DOTA-PEG_11_-Tz rapidly cleared with poor *in vivo* performance. [^177^Lu]Lu-DOTA-PEG_7_-Tz exhibited the highest tumor uptake, which significantly slowed tumor growth compared to vehicle-injected mice or [^177^Lu]Lu-DOTA-PEG_7_-Tz alone. Interestingly, despite the promising *in vivo* results and the availability of the Tz/TCO click chemistry reagents, this strategy has not yet been applied in clinical trials for either imaging or RIT purposes at the time of this writing 97. However, it is worth noting that a phase I trial (NCT04106492) examining the potential of Tz/TCO click chemistry strategy using a biopolymer-Tz conjugate to target TCO-bound doxorubicin (estimated study completion date Aug. 2023) is currently underway. This can potentially pave the way for the translation of pRIT to clinical trials. Other click chemistries such as avidin/biotin binding have also been utilized to target the radionuclide to the antigen-bound mAb, however, given their immunogenicity, clinical translation remains a challenge 98,99.

## Radionuclide carrier platforms

### Full-length mAbs

The primary role of mAbs for RIT is to direct the therapeutic radionuclide payload to the tumor by targeting and binding onto antigens located on the surface of cells with high specificity. This can result in the internalization of the antibody:antigen formed complex. A brief discussion on the benefits of internalization is provided further below. The commercial availability of full-length immunoglobulins has made them the primary choice for the development of the majority of RITs. For example, the full-length mAb 376.96, which recognizes an epitope of B7-H3 that is expressed on ovarian cancer and cancer initiating cells, has been labeled with ^212^Pb for targeted α-particle therapy in intraperitoneal (i.p.) models of human ovarian cancer xenograft models, ES-2 and A2780cp20. Animals treated with the RIT demonstrated a two- to three-fold longer survival compared to control groups 100.

Receptor tyrosine kinases are attractive targets for RIT agents. Disseminated peritoneal tumors were treated with ^212^Pb radioimmunoconjugates specific for EGFR or HER2 101-103. Other studies employed panitumumab as carriers of different radioisotopes such as ^177^Lu, ^90^Y and ^111^In (Table 2) targeting different EGFR^+^ tumors 104-106. One unique study with panitumumab examined treatment of PANC-1 tumors with ^177^Lu labeled to metal-chelating polymers (MCP) bound to 13 DOTA chelators 107. The organs that received the highest absorbed dose of RIT in the study were the pancreas (19.3 Gy), kidneys (15.7 Gy), spleen (14.8 Gy) and liver (7.5 Gy). The absorbed doses in the tumor averaged 12.3 Gy with no renal or hepatic toxicity observed. Several other reports examined the chimeric anti-EGFR antibody cetuximab as a RIT agent using ^188^Re, ^177^Lu, ^64^Cu and ^212^Pb (Table 2) 105,108-113.

A head-to-head comparison conducted by Liu *et al.* compared panitumumab and cetuximab and their ^177^Lu-labeled counterparts in a UM-SCC-2B human head and neck squamous carcinoma tumors 105. Their findings revealed no inhibitory effects were observed in both non-labeled panitumumab or cetuximab-treated cohorts. However, a 14.8 MBq dose of both ^177^Lu-labeled mAbs resulted in significantly delayed tumor growth. This effect was attributed to ^177^Lu with only ~10 µg of each of the mAbs administered in the RIT study when compared to tumor response in the cohorts treated with ~200 µg of the naïve antibodies. Interestingly, [^177^Lu]Lu-DOTA-panitumumab exhibited a stronger anti-tumor effect, with almost complete response at 36 d post treatment versus [^177^Lu]Lu-DOTA-cetuximab, where tumors relapsed after 30 d. Although [^177^Lu]Lu-DOTA-cetuximab was observed to have higher EGFR-binding affinity, the therapeutic effect was postulated to stem from better tumor penetration of [^177^Lu]Lu-DOTA-panitumumab due to a “binding site barrier” effect which will be discussed in later. It is also worth noting that cetuximab and panitumumab have differing IgG subtypes (IgG1 and IgG2, respectively) which may also contribute to the difference in affinity and subsequent tumor accumulation 114. Taken together, these studies highlight tumor penetration as key in RIT efficacy.

A notable study demonstrated the usefulness of the Fc region of full-length mAbs. The full-length hu11B6 antibody labeled with either ^225^Ac- or ^177^Lu targeted the catalytically active enzyme human kallikrein 2 (hK2) in prostate cancer 115-117. The formed hu11B6:hK2 complex is internalized via Fc:Fc receptor (FcRn) mediated intracellular uptake. This is then trafficked to the lysosome for processing, which brings the therapeutic radionuclides within sufficient proximity to the nucleus to render a therapeutic effect 118.

### Antibody Fragments

Although the use of full-length mAbs for RIT development is popular, there are several drawbacks. Notably, full-length mAbs inherently have a longer blood circulating time, decreased vascular permeability and lower diffusivity within solid tumors. These challenges led to the exploration of mAb fragments as radionuclide vectors. Mab fragments are deemed to have better favorable pharmacokinetics (PK) and tumor penetration profiles as RIT agents due to their small molecular size and lack of Fc 121. Mab fragmentation and engineering can alter the main clearance route of tracers from the liver to the kidney. This can further affect the dose limiting tissue and potentially the effective dose. The change in clearance routes is a result of kidney glomerular filtration of molecules below ~60-70 kDa in size. Larger molecules are observed to clear through the liver 122.

Full-length mAbs can either be enzymatically cleaved into F(ab')_2_ (~110 kDa) or Fab fragments (50-55 kDa). MAbs can also be genetically engineered to produce scFv-Fc (105 kDa), minibodies (80 kDa), diabodies (55 kDa), single chain variable fragments (scFv, 28 kDa), or single domain antibodies (sdabs, 12-15 kDa), which are the variable domain of heavy-chain antibodies (V_H_H) also called nanobodies 81,123,124. Table 3 provides a partial list of the most commonly used antibody platforms.

The studies discussed in this section focused only on three prominently explored targets - EGFR, HER2 and CEA primarily due to the wide body of work investigating different antibody formats specific to these RIT targets. This allows a facile comparison of effects in size, radionuclide, chelate, PK and effective dose.

Smaller fragments have been shown to mitigate the drawback of hematological toxicity resulting from the long circulating t_1/2_ of full-length mAbs. These fragments are associated with increased clearance rates from blood and normal tissues. The trade-off to improving clearance is a lower intratumoral absorbed dose as a result of the fragments' rapid clearance 6,57. Generally, smaller fragments may possess lower affinities, combined with increased blood clearance kinetics, which can result in lower absolute tumor uptake 57. However, it has also been noted that smaller fragments (<55 kDa) may lead to more rapid tumor accumulation as a result of increased diffusion into the tumor 121. Moreover, the selection of a mAb-carrier for RIT extends beyond circulating t_1/2_ and blood toxicities and is governed by a number of variables including the Thiele modulus, vascular permeability, diffusivity, affinity, valency, specificity and tumor retention.

The Thiele modulus refers to characteristics of mAb internalization and lysosomal degradation. It is a ratio determined by the internalization rate to the diffusion or binding rate. The modulus favors an increased diffusion rate which should allow for a more homogenous intratumoral distribution of the RIT agent 121. Although the size of the mAb plays a significant role in the Thiele modulus concept, smaller fragments are preferred due to fast tissue clearance, reducing normal tissue exposure.

Examining the occurrence of internalization and its kinetics is a prerequisite for each RIT agent to follow the fate of the antibody:antigen complex. If proven to internalize, utilizing a therapeutic radiometal would be beneficial since it is residualized, or retained intracellularly 128. A study in peritoneal carcinomatosis compared the efficacy and toxicity of ^212^Pb-labeled mAbs that target either HER2 using trastuzumab, which is known to internalize, or CEA using 35A7 mAb, a non-internalizing mAb 129. Mice with i.p. tumors treated with [^212^Pb]Pb-34A7 had a median survival (MS) of 94 d, whereas the MS of mice treated with [^212^Pb]Pb-trastuzumab, was not determined since >50% of the mice in this cohort survived past 130 d. Taken together, this study underscores the benefit of an internalizing antibody:antigen complex. This key feature is critical in AE therapy as its short path length requires proximity to nuclear DNA to have therapeutic effect 130.

The vascular permeability of a RIT agent is inversely associated with its size wherein smaller agents have increased vascular permeability. Aberrant tumors with a hypervascularized nature can potentially increase the drug's penetration beyond the tumor periphery 131. As a result, molecules that are >40 kDa (e.g. full-length mAbs, F(ab')_2_, Fab, minibodies and diabodies) can extravasate into a tumor, irrespective of specific targeting. This phenomenon, known as enhanced permeability and retention (EPR) effect, results from a combination of poor lymphatic drainage and increased blood circulating t_1/2._ Higher retention of macromolecules is observed as a consequence. However, the EPR effect does not necessarily increase the therapeutic efficacy of tracers 121. The interstitial fluid pressure (IFP) is also increased by poor lymphatic drainage, which results in an inverse correlation between the diffusion of macromolecules into the tumor interstitium and their size.

Beyond extravasation, a RIT agent requires sufficient diffusivity for homogenous distribution into the tumor. The diffusivity of mAbs, or their fragments, is driven by the intercellular concentration gradient, which can be directly affected by the extracellular matrix (ECM) including the concentration of collagen 121. Overall, it has been shown that smaller forms of mAbs exhibit increased diffusivity 132. A successful RIT agent should have sufficient tumor retention, diffusion and affinity for its target. For high affinity binding mAbs, the “binding site barrier effect” suggests that intratumoral penetration of antibodies can be inhibited by their size and high affinity for the antigen 133,134, as observed in the aforementioned therapeutic efficacy noted between [^177^Lu]Lu-DOTA-panitumumab and [^177^Lu]Lu-DOTA-cetuximab 105. Collectively, the interplay of these concepts is an imperative consideration of the antibody platform choice to produce beneficial results in RIT development.

### Nanobodies

The nanobody is one of the smallest forms of engineered antibodies with a size of 12-15 kDa (Table 3). Nanobodies are derived from the naturally occurring subtype of antibodies, known as heavy chain antibodies (HCabs), that are found in camelids. These HCabs do not possess light chains, resulting in the absence of the first constant domain. The antigen binding portion of HCabs contains only one single variable domain, or VHH, that is known as a single-domain antibody (sdAb) or nanobody 135. The HER2-specific nanobody, 2Rs15d, has been labeled with ^177^Lu and its treatment efficacy was compared against [^177^Lu]Lu-DTPA-trastuzumab in a HER2-expressing xenograft model. [^177^Lu]Lu-DTPA-2Rs15d resulted in quick clearance of non-target tissues with an observed higher uptake in the tumor compared to the kidneys beyond 24 h p.i. Maximum uptake in the kidneys was noted at 48 h p.i., but remained >4-fold lower than the tumor. While the intratumoral uptake of [^177^Lu]Lu-DTPA-trastuzumab was six-fold higher, the radiation dose delivered to off-target organs was significantly elevated (spleen: 80-fold, bone: 26-fold and blood: 4180-fold higher) 36,37. The same nanobody was labeled with ^225^Ac and co-injected with Gelofusin, a plasma extender, which appreciably reduced renal uptake in HER2^+^ SKOV-3 xenografts. Albeit, a slight decrease in tumor uptake was observed 20. A separate study by the same group demonstrated accumulation of [^225^Ac]Ac-DOTA-2Rs15d in intracranial SKOV-3, whereas labeled trastuzumab did not accumulate in a similar but separate cohort. Moreover, the co-injection of trastuzumab with [^225^Ac]Ac-DOTA-2Rs15d improved the median survival of mice compared to treatment with trastuzumab alone 136.

### Diabodies

Diabodies (~55 kDa) are formed when two single chain Fvs are linked together via a peptide chain (Table 3) and exhibit a serum t_1/2_ around ~5 h 125,137. Investigators have shown that an ^90^Y-labeled CHX-A”-DTPA-C6.5K-A diabody targeting HER2 was effective at inhibiting growth rates of MDA-361/DYT2 breast tumor xenografts. Of note, even though higher tumor accumulation was observed with the diabody, this comes with the penalty of greater absorbed doses to off-target tissues compared to the [^177^Lu]Lu-DTPA-2Rs15d nanobody 37,138. Table 4 lists all RIT agents targeting HER2 using different mAb-formats, radionuclide, chelator, dose administered, study information and tumor model. Although majority of the noted studies included combination therapies, different dosing schedules and tumor types, one notable trend is the increase in administered activity as the antibody format size decreases, to augment decreased tumor accumulation stemming from rapid clearance rates.

### Fab fragments

An antigen binding fragment or Fab (~50-55 kDa) is derived from a full-length IgG via enzymatic or chemical digestion (Table 3). The cleavage of the Fc region shortens its blood circulation, exhibiting a serum t_1/2_ of 12-20 h 125. A study by Razumienko *et al*. examined a bispecific radioimmunoconjugate developed from the Fab region of trastuzumab linked with epidermal growth factor (EGF) ligand through a PEG_24_ spacer 139. The bispecific radioimmunoconjugate provides the benefit of targeting tumors that co-express HER2 and EGFR. [^177^Lu]Lu-DOTA-Fab-PEG_24_-EGF displayed higher tumor accumulation (7.3 ± 1.5 %ID/g) than just the radiolabeled trastuzumab Fab (3.2 ± 1.5 %ID/g) and EGF ligand (2.1 ± 0.7 %ID/g) alone. This study further highlighted the comparison between the bispecific immunoconjugate and the full-length mAb, noting that the hematologic toxicity of [^177^Lu]Lu-DTPA-trastuzumab was significantly higher than the bispecific radioimmunoconjugate (13.7 ± 0.8 %ID/g vs. 1.2 ± 0.7 %ID/g, respectively). This is not surprising as bispecific [^177^Lu]Lu-DOTA-Fab-PEG_24_-EGF can clear more rapidly from the blood due to its size.

### F(ab')_2_

F(ab')_2_ fragments (~110 kDa) are a derivative of the intact IgG whereby the Fc region is removed through enzymatic cleavage with pepsin leaving two antigen binding fragments linked via disulfide chains (Table 3). Like its parent mAb, this fragment has been utilized to target different cell-surface antigens, but with a shorter t_1/2_ of ~8 - 10 h 125. The anti-L1-CAM antibody chCE7 was fragmented to a F(ab')_2_ construct then labeled with either ^177^Lu or ^67^Cu 42. Both RITs exhibited similar tumor uptake with higher renal uptake than the intact mAb. However, the *in vivo* biodistributions differed between the two radiometals with lower kidney uptake for the ^67^Cu RIT agent. The difference was thought to be from the negative charge of the [^67^Cu]Cu-DOTA, compared to the neutral charge of the [^177^Lu]Lu-DOTA complex. Nonetheless, the ^67^Cu-labeled chCE7 F(ab')_2_ seemed more promising with two-fold enhancement in the tumor-to-kidney ratios. Unfortunately, tumor response and survival rates were not examined for this study.

A ^212^Pb-labeled F(ab')_2_ fragment derived from panitumumab demonstrated a survival advantage compared with animals treated with ^212^Pb-labeled nonspecific F(ab')_2_ in a model of abdominal carcinomatosis 119. In a separate study, RIT with [^64^Cu]Cu-NOTA-panitumumab F(ab')_2_ as a monotherapy of PANC-1 pancreatic cancer xenografts in mice was unsuccessful as tumor doubling time and median survival were not affected 120. However, when combined with gemcitabine and the PARP inhibitor rucaparib, a significant median survival benefit was observed compared to RIT alone or gemcitabine and rucaparib, highlighting the benefit of RIT development with combination therapies. Another study using a F(ab')_2_ fragment of cetuximab, [^177^Lu]Lu-DOTAGA-F(ab')_2_-cetuximab, resulted in decreased tumor growth for colorectal xenografts in nude mice at 2, 4 and 8 MBq compared to vehicle control, with significantly decreased tumor volumes in the 4 and 8 MBq administered cohorts 112.

Overall, these studies demonstrate the breadth of RIT development. Unsurprisingly, opinions on the antibody platform choice for RIT development have varied. Wittrup *et al*. noted that IgG antibodies would be the optimal size for tumor uptake, based on vascular permeability, the clearance t_1/2_, passive renal clearance and intravenous injection 144. Others postulate that smaller size fragments are superior with respect to circulation time, tissue penetration, and homogeneous distribution 128,145. Ultimately, each antibody format, and antibody-target pairing, requires proper characterization and preclinical evaluation to determine the effective format and dose as modifications on size affect uptake, therapeutic efficacy and dose.

## Other emerging strategies to modulate tumor delivery and PK

Several engineered mAb formats and methods have emerged in attempts to modulate PK. Utility of a one-armed monovalent mAb (99 kDa) (e.g. onartuzumab) displayed altered clearance via the kidneys instead of hepatic elimination 146. Other approaches incude a scFv-Fc fusion protein, which is a fragment (105 kDa) that utilizes the scFv as a building block, engineered with the full Fc region. Coupling of Fc to scFv mitigates rapid renal clearance that is observed with fragments below the renal glomerular filtration threshold, potentially increasing the absorbed dose to the target. An immunoPET imaging study using an anti-CEA T84.66 scFv-Fc with five different mutations in the Fc region confirmed the utility of this strategy 126. To the best of our knowledge and at the time of writing, only one published study reported on utilizing a scFv-Fc fusion protein for RIT development targeting the tumor endothelial marker-1 (TEM-1) 147. [^177^Lu]Lu-DOTA-1C1m-Fc was evaluated in TEM-1 positive human neuroblastoma (SK-N-AS) tumors compared to TEM-1 negative human fibrosarcoma (HT-1080) tumors. Although this study only confirmed a 1.9-fold increase in uptake in TEM-1 positive tumors, compared to negative controls, it established the potential applicability of an scFv-Fc engineered antibody for RIT development. Further to using scFv-Fcs, a separate study tuned the serum half-life by introducing a single point mutation in the C_H_2 domain of the Fc region of an anti-CA19.9 scFv-Fc. The mutation lowered the blood pool residency, which can potentially be exploited for RIT development as a means to mitigate hematologic toxicity 148.

## Preclinical evaluation

### MTA and Organ Radiosensitivity

Pharmacological considerations in the preclinical development of effective RITs, including the selection of radionuclide, are centered around the *in vivo* tolerance of the activity administered, tumor size, antigen homogeneity/heterogeneity, exposure of radiosensitive organs and establishing a therapeutic index (TI) 15. The goal of the TI is to increase the ratio of the absorbed activity to the tumor compared to normal tissue, which to date, remains a challenge in RIT of solid tumors 98,128. Under ideal circumstances, the TI of the tracer in the tumor would be infinite with no off-target tissue absorbed doses. This would yield tolerance of the RIT agent with minimal to no significant adverse effects. Generally, the organs at risk for toxicity include the bone marrow, kidney, lungs and colonic mucosa 57,128,149. The TI ideally should be >50-fold for bone marrow and >10-fold for kidney 128. To examine adverse effects, separate cohorts of mice are typically treated with increasing activities of the RIT agent to establish the MTA. The MTA has also been described as the maximum tolerated dose (MTD) administered and reported as mg/kg of the RIT agent. MTA determination is critical to identify an optimum single dose and/or establish dosing schemes at or below the MTA threshold 150. It is important to note that the MTA/MTD may not be efficacious. Therefore, optimization of the drug's efficacy, safety and tolerability by altering its PK or administration schedules (e.g. fractionation), can achieve the desired therapeutic effect while minimizing toxicities 29.

To monitor the rodents' overall health following RIT administration, some studies monitored clinically applicable parameters of RIT including markers of liver function (alkaline phosphatase, aspartate transaminase, alanine transaminase), kidney function (urea, creatinine), and extent of bone marrow suppression (WBCs, RBCs, hemoglobin, platelets) 107,120,139,151. For example, Razumienko *et al.* examined the bispecific [^177^Lu]Lu-DOTA-Fab-PEG_24_-EGF for HER2 and EGFR, and noted decreases in RBCs, WBCs and hemoglobin in the 18.5 MBq dose cohort compared to the untreated, 3.7 MBq and 11.1 MBq cohorts, suggesting hematologic toxicity 139. Creatinine and alanine aminotransferase levels were not significantly affected, indicating that kidney and liver toxicities were not prevalent at any administered activity, respectively. These results led the authors to choose 11.1 MBq for RIT studies as no adverse effects were observed. Similarly, α-particle RIT targeting glypican-3, which is expressed in liver cancers, demonstrated significant hematologic toxicity and minimal acute liver or kidney toxicity. Notably, authors did not assess any late effects on these organs 26. These findings are consistent with those observed in clinical trials 9-11,152.

### Route of Administration

The traditional route of tracer administration is via intravenous (i.v.) injection, where the tracer systemically circulates through the blood until it extravasates into the tumor tissue or clears through the liver or kidney. Altering the route of administration can be advantageous as locoregional administration of the agent can immediately target lesions within close proximity. As an example, a tracer injected through the peritoneum (i.p.) likely has increased tumor accumulation in peritoneal disseminated tumors (e.g. carcinomatosis) than when administered systemically. In one study, mice bearing orthotopic xenografts of xPA-1-DC pancreatic cancer cells (xPA-1) were treated i.p. with adjuvant [^64^Cu]Cu-PCTA-cetuximab or conventional adjuvant gemcitabine following surgical resection versus surgical resection-only cohorts. The mice treated with [^64^Cu]Cu-PCTA-cetuximab experienced prolonged survival compared to those treated with adjuvant gemcitabine or the surgically resected mice 110. The i.p. route of administration was considered advantageous as it limits exposure to normal tissues, and only targets the tumor. Additionally, clinical trials have also examined the benefit of an i.p. route of administration to complement i.v. administration 102,103. An aforementioned pRIT study employed multiple administration routes combining pRIT with i.v. and i.p. administration of [^177^Lu]Lu-DOTA-Tz with a TCO-conjugated anti-carcinoembryonic antigen (CEA) antibody 35A7 targeting peritoneal carcinomatosis of colorectal origin 96. Between i.v. and i.p. administration, no significant influence on biodistribution of the tracer was observed. Thus, the study utilized i.v. injection of 35A7-TCO in combination with i.p. of [^177^Lu]Lu-DOTA-Tz. Milenic *et al.* explored the combination of i.v. and i.p. injected ^212^Pb-labeled F(ab')_2_ fragment of panitumumab in LS-174T tumor-bearing mice. While treatment via combined i.p. and i.v. administration may be effective, further studies are warranted as the benefit of combination administration has been discordant 119. Moreover, studies of radioiodinated antibodies have shown benefit following combined i.p. and i.v. administration, while others had no advantage 153,154.

An exploration of intra-compartmental administration can also be considered for tumors restricted to an accessible body cavity. The benefit of intra-compartmental administration may prevent dilution of the RIT by increasing radiation-absorbed doses by 10-fold compared to i.v. injections. For example, intrathecal administration distributes the RIT in the cerebral spinal fluid (CSF) in a smaller overall volume (~150 mL total volume) compared to intravenous administration (~5 L). Additionally, CSF flows in one direction and is replenished after seven to eight hours, providing a washout of the antibody. Finally, there are no WBCs or proteins present in CSF that could affect antibody binding. To date, intrathecal and intraventricular administration of RIT have only been examined using non-metal radionuclide labeled mAbs for primary brain tumors or leptomeningeal disease 155-158.

pRIT has been explored as an alternative approach to administer RIT agents to increase TI, as previously mentioned in the click chemistry section. The two-step approach of pRIT decouples the antibody from the radionuclide to mitigate the toxicity effects observed with a circulating RIT 128. The pRIT employs a non-radiolabeled immunoconjugate that can target both a tumor-specific cell surface antigen and the radioligand. First, the immunoconjugate is administered to target the cell surface antigen and clear from nonspecific sites. Subsequently after a period of time, the therapeutic radionuclide is administered 159. The small size of the radiometal-chelate complex can rapidly target and bind to cell-surface bound mAb. An understanding of the antibody-antigen binding kinetics is necessary to fully realize the benefits of pRIT. Internalization of the tumor antigen-bound mAb must be minimal to allow for the radiometal complex to “click” to the mAb, otherwise tumor response can be negatively affected 160. In some approaches, a final step utilizing a clearing agent to further enhance bloodpool clearance of the radionuclide is included in the treatment scheme 128.

**Figure 3:**
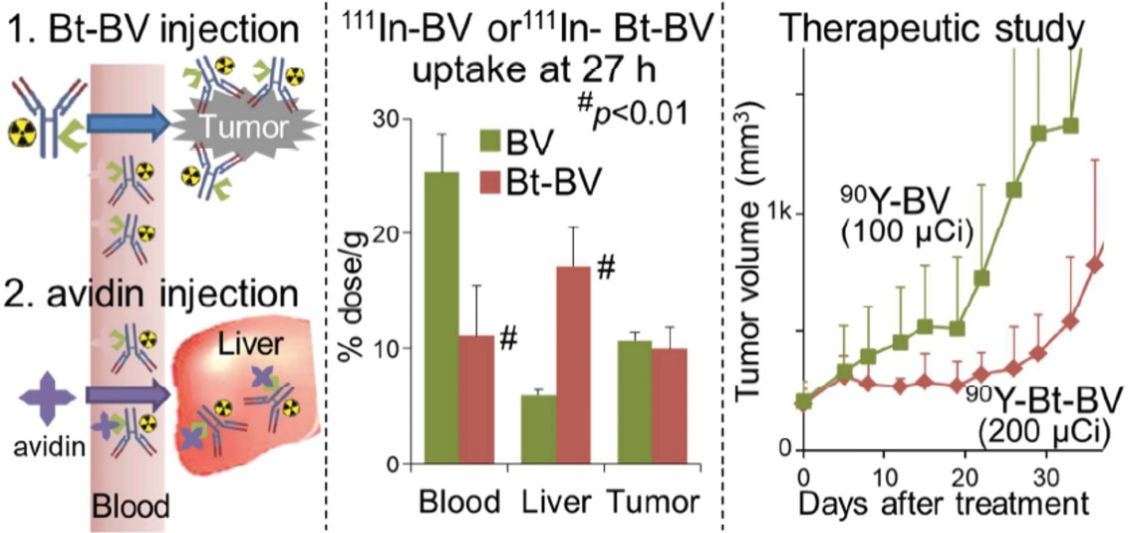
** Left Panel:** Schematic of Biotin-Bevacizumab injection followed by an avidin chase. **Middle Panel:** [^111^In]In-DTPA-Bv and [^111^In]In-DTPA-Bt-Bv were utilized to examine the biodistribution profiles of the tracer 27 h post injection. [^111^In]In-DTPA-Bt-Bv exhibited significantly lower blood uptake and higher liver uptake. **Left Panel:** In the therapeutic study, the [^90^Y]Y-DTPA-Bt-Bv cohort of mice was significantly inhibited compared to the [^90^Y]Y-DTPA-Bv. Adapted with permission from Yudistiro et al., Molecular Pharmaceutics, 2018. Copyright 2018 American Chemical Society.

An alternative modification to pRIT is a “chase” injection strategy in which a radiolabeled biotinylated-mAb is injected, followed by an avidin “chase” to accelerate blood clearance. A study utilized this chase strategy by injecting avidin following administration of the ^90^Y-labeled, biotinylated anti-VEGF mAb bevacizumab to target triple-negative breast cancer (Figure 3). This unique approach cleared excess antibody and increased the MTA of [^90^Y]Y-DTPA-biotinylated-bevacizumab in non-tumor-bearing mice from 9.5 MBq to 11.1 MBq and in tumor-bearing mice from 3.7 MBq to 7.4 MBq 161. The increase in MTA allowed for a two-fold increase in administered [^90^Y]Y-DTPA-biotinylated-bevacizumab (200 μCi) compared to the non-biotinylated RIT (100 μCi) resulting in suppressed tumor growth. Although the biotin-avidin pre-targeting strategy is utilized preclinically for proof-of-concept studies, clinical translation is challenging due to the immunogenicity of avidin 96.

### Fractionation

**Figure 4:**
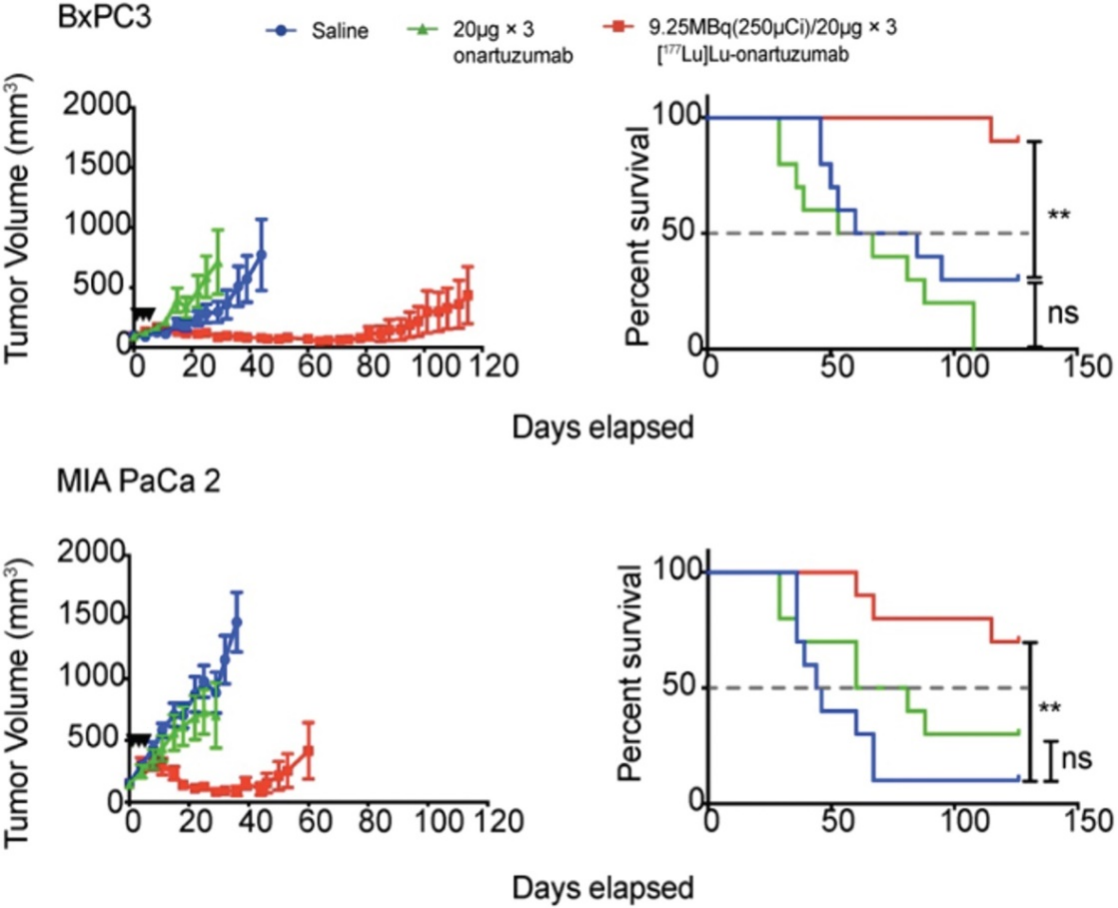
High MET-expressing BxPC3 and low MET-expressing MIA PaCa 2 tumors were treated with a fractionation schedule 9.25 MBq/20 µg of [^177^Lu]Lu-DTPA-Onartuzumab. The fractionated schedule showed therapeutic efficacy for both high and low MET expressing tumors compared to saline and non-labeled onartuzumab controls. Adapted with permission from Escorcia et al., Theranostics, made available under a Creative Commons CC-BY license.

Fractionation divides a therapeutic dose over numerous smaller doses or “fractions”, and, is well-established for external beam radiotherapy. Because this approach can maximize total dose to the target tissues while minimizing radiation toxicity of normal radiosensitive tissues, fractionated systemic administration of radioactivity continues to be investigated for efficacy and improved safety benefits. In this setting, fractionation can increase the total absorbed dose to the target tissues by providing multiple doses at, or below, the MTA/MTD 162. Fractionation has been preclinically examined in pancreatic ductal adenocarcinoma with [^177^Lu]Lu-DTPA-onartuzumab, a one-armed anti-Met antibody. The study, which examined various pancreatic ductal adenocarcinoma (PDAC) cell lines with differing Met expression levels, demonstrated that membrane dynamics is essential in the development membrane receptor targeted therapeutics. After showing that Met is recycled back to the membrane following treatment with onartuzumab, the study deemed that a fractionated dosing schedule is more favorable since it maximized accumulation of the RIT agent within the tumor as opposed to a bulk, single dose injection. Mice were treated with 9.25 MBq every 72 h for three doses of [^177^Lu]Lu-DTPA-onartuzumab and showed a significant overall survival coupled with significant tumor growth delay (Figure 4) 146. Conversely, a study examining fractionated doses of [^64^Cu]Cu-DOTA-trastuzumab combined with paclitaxel provided no therapeutic efficacy for mice with HER2-positive gastric cancer xenografts 163. Thus, the mixed results of fractionated administration underscore the need for further preclinical and clinical investigation.

Of note, the combination of administration routes and dosing schemes may provide a survival benefit following treatment. For example, Cheal *et al*. examined fractionated pre-targeted radioimmunotherapy for HER-2 positive BT-474 breast cancer xenografts 71. The study utilized a bispecific IgG-scFv format antibody (210 kD) targeting HER2 with the IgG sequence of trastuzumab and the scFv C825, which has high affinity for Bn-DOTA. A comparison of a single dose treatment of 55.5 MBq of [^177^Lu]Lu-DOTA-Bn to fractionated treatment of three doses of 55.5 MBq (total of 167 MBq) administered once weekly was made. They observed a size-dependent response in which very smaller tumors (< 30 mm^3^) required a single dose to exhibit a complete response (estimated tumor absorbed dose 22 Gy) whereas the medium-sized tumors (209±101 mm^3^) required three treatments for a complete response (estimated tumor absorbed dose 66 Gy). These findings suggest that personalized dosimetry may be important for optimizing anti-tumor responses in the clinic.

### Merging RIT with other therapies for optimized efficacy

RIT monotherapy alone does not always produce favorable survival results. Combining treatment with radiosensitizers in tumors can potentially enhance tumor response and patient outcomes 164. Radiosensitizers are often non-toxic to normal cells but are utilized to improve the therapeutic efficacy of the RIT. Chemotherapeutics such as gemcitabine and paclitaxel have been shown to improve the efficacy of ^212^Pb-labeled trastuzumab 113,165. Additionally, emerging radiosensitizers such as PARP inhibitors (PARPi) like rucaparib have been examined in combination with gemcitabine and [^64^Cu]Cu-NOTA-panitumumab F(ab')_2_ 120. As previously noted, the addition of the radiosensitizers significantly improved the median survival. The ATRi inhibitor BAY 1895344 also improved efficacy of the ^227^Th-labeled fibroblast growth factor receptor 2 mAb (FGF2) BAY 1179470 in the MFM-223 breast cancer xenograft model 166.

RIT was also combined with agents that enhance perfusion, modulate surface receptors and tyrosine kinase signaling pathways, damage DNA and inhibit immune checkpoint. As previously noted, Puttemans *et al.* determined that a combination therapy strategy of [^225^Ac]Ac-DOTA-2Rs15d and trastuzumab in the SKOV3.IP1 cohort increased median survival by 12.5 days 136. Beyond antibodies as combination therapeutics, the efficacy of [^177^Lu]Lu-DOTA-chCE7 combined with protein kinase inhibitors (PKIs: alisertib, MK1775, MK2206, saracatinib, or temsirolimus) was examined in SKOV3ip and IGROV1 ovarian cancer xenografts. Ultimately MK1775 (AZD1775), an inhibitor of Wee1 tyrosine kinase, in combination with [^177^Lu]Lu-DOTA-chCE7 produced more DNA double strand breaks in SKOV3ip and decreased the tumor growth for IGROV-1 167. Combination therapy was further examined in BxPC-3 pancreatic cancer with ^90^Y-labeled 059-053 mAb targeting CD147 and gemcitabine in one- and two-cycle regimens 168. Both cycles produced favorable tumor growth inhibition responses, however, the two-cycle group of mice experienced severe adverse effects.

[^177^Lu]Lu-DOTA-PEG-scVEGF targeting VEGFR also utilized in a combination treatment strategy with the antiangiogenic drugs bevacizumab or sunitinib. [^177^Lu]Lu-DOTA-PEG-scVEGF was administered at the lowest effective dose (7.4 MBq/mouse) as a pretreatment to disrupt tumor vasculature and inhibit orthotopic breast cancer tumor growth, followed by either bevacizumab or sunitinib. Pretreatment with the RIT agent followed by bevacizumab or sunitib significantly slowed tmor growth than single agent bevacizumab or sunitinib 169.

### Companion Diagnostics

**Figure 5:**
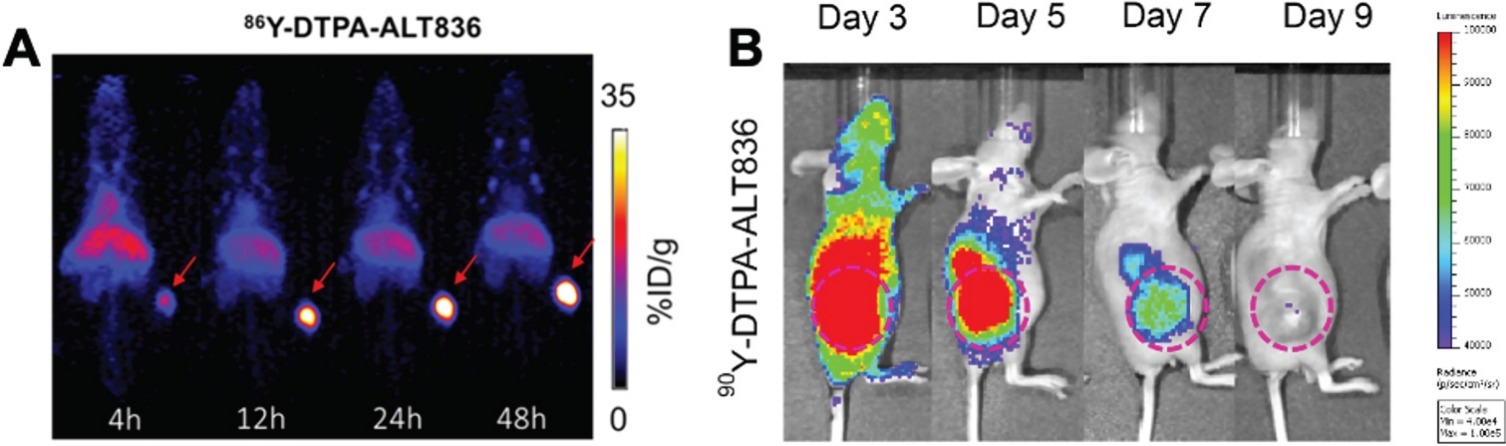
(**A**) ImmunoPET and (**B**) Cerenkov luminescence imaging of mice injected with ^86^Y-labeled and ^90^Y-labeled ALT836, respectively. The high uptake of tracer in the tumor visualized by immunoPET is recapitulated with high uptake of the RIT observed by Cherenkov luminescence imaging. Adapted with permission from Ferreira, C.A., et al. ^86^/^90^Y-Labeled Monoclonal Antibody Targeting Tissue Factor for Pancreatic Cancer Theranostics. Mol. Pharm. **2020**, 17 (5), 1697-1705. Copyright *2020 American Chemical Society.*

The ease of modifying radiolabeled antibodies by swapping out the imaging radionuclide for a therapeutic one has led to the development of numerous “theranostics”, to image then treat malignancies 146. These theranostics can be beneficial as imaging can interrogate antigen expression, inform on tracer localization to target tissue, organ biodistribution/dosimetry and the potential for a response to therapy, prior to RIT. For hepatocellular carcinoma, two studies have evaluated RIT response with yttrium-90 labeled mAbs. The first study utilized [^111^In]In-DOTA-anti-ROBO1 for biodistribution and [^90^Y]Y-DOTA-anti-ROBO1 for therapy. The theranostic initially showed significant inhibition of HepG2 tumor growth in mice, but tumors regrew at day 20 38. An examination of the biodistribution of [^111^In]In-DOTA-anti-ROBO1 suggested a maximum uptake of tracer in the tumor at 48 h p.i., which decreased until the final time point examined at 240 h p.i. This decrease in tracer uptake coupled with ^90^Y decay may explain why tumors relapsed at 20 d after treatment. Labadie *et al.* demonstrated that ^89^Zr immunoPET imaging can successfully assess tumor response to glypican-3 targeted ^90^Y RIT 170. In pancreatic cancer, Ferreira *et al.* targeted tissue factor (TF), which is overexpressed in various malignancies, with ^86^Y-labeled ALT836 mAb for imaging and [^90^Y]Y-DTPA-ALT836 for therapy of BxPC-3 xenografts resulting in increased survival (Figure 5) 39.

A seminal study tested the theranostic potential of hu5A10, a humanized mAb targeting free prostate-specific antigen (KLK3), in prostate cancer 171. Pharmacokinetic properties of [^89^Zr]Zr-hu5A10 in non-human primates showed similar profiles in mice. A head-to-head comparison of ^90^Y and ^225^Ac labeled hu5A10 demonstrated immediate but unsustained response in mice (1/9) treated with [^90^Y]Y-hu5A10. Complete responses were noted in 7/18 mice treated with the [^225^Ac]Ac-hu5A10.

## Updates on Clinical Trials

Metals-based RIT clinical trials have grown owing to the potential efficacy that were realized in preclinical studies. A phase I trial (NCT01384253) of i.p. administered [^212^Pb]Pb-TCMC-trastuzumab showed promise in patients with HER2 expressing ovarian cancer 102,103. The initial study examined the pharmacokinetic results after administration of 7.4 MBq/m^2^ i.p. following a 4 mg/kg i.v. infusion of trastuzumab. This small study (n=3) identified a tolerable response with <6% of the tracer observed outside the peritoneal cavity. A follow-up study in this trial examined 16 patients with HER2 expressing malignancies (fifteen ovarian cancer patients, and one male with HER2^+^ colon cancer) with disease relapse 102. The study investigated five activity levels between 7.4 and 21.1 MBq/m^2^ with n = 3-4 patients/cohort and determined that there were minimal toxicities at >1 year of treatment for the 7.4 MBq/m^2^ cohort and at >4 months for the subsequent cohorts.

In a phase I trial, [^90^Y]Y-DOTA-M5A was combined with gemcitabine for patients with chemotherapy refractory metastatic CEA-producing malignancies 40. The anti-CEA antibody had benefit over previously explored antibodies because the humanized nature prevents the development of human anti-murine antibody (HAMA) responses 172,173. Although patients initially received gemcitabine, grade 3 thrombocytopenia and leukopenia developed in three of the first four patients causing its removal from the trial.

**Figure 6:**
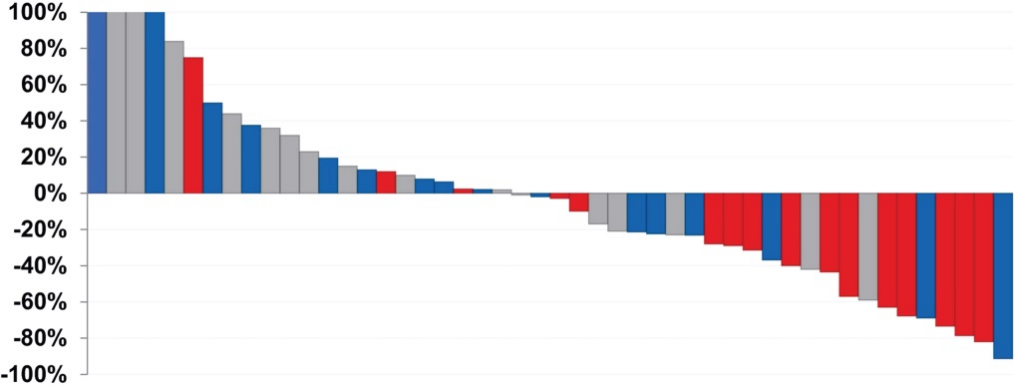
Waterfall plot demonstrating PSA response of patients treated with varying doses of [^177^Lu]Lu-J591. Gray: 20-35 mCi/m^2^ × 2, Blue: 40 mCi/m^2^ × 2, Red: 45 mCi/m^2^ × 2. As the doses increased, the PSA response decreased. Adapted with permission from Nanus *et al.* Phase 1/2 study fractionated dose lutetium-177-labeled anti-prostate-specific membrane antigen monoclonal antibody J591 (^177^Lu-J591) for metastatic castration-resistant prostate cancer. *Cancer*. **2019**, 125 (15), 2561-2569.

Patient response to radionuclide therapy targeting anti-prostate-specific-membrane-antigen (PSMA) has been extensively explored. At the time of this review, seven clinical trials were conducted utilizing J591, a PSMA-specific full-length mAb labelled with ^177^Lu. The initial phase I single dose escalation trial identified a 70 mCi/m^2^ MTA 174. The phase II single dose study (NCT00195039) assessed the efficacy of treatment with 65 mCi/m^2^ and 70 mCi/m^2^ and confirmed a PSA response and increased survival from 11.9 months to 21.8 months 175. An independent phase I/II trial examined low (40 - 70 mCi/m^2^) versus high (80 - 90 mCi/m^2^) cumulative fractionated doses in metastatic castrate resistant prostate cancer (mCRPC) (NCT00538668). Results of the trial demonstrated that higher cumulative doses of [^177^Lu]Lu-J591 can be delivered in a fractionated schema. The higher doses corresponded to a decrease in prostate serum antigen (PSA) (Figure 6). The dose limiting toxicity stems from myelosuppression, albeit tolerable, owing to the long blood pool kinetics of J591. However, the authors viewed prolonged circulation of the RIT agent as an advantage with continuous tumor delivery to the tumor spread out over several days 162.

A separate phase I dose escalation study (NCT00916123) tested the combination of docetaxel, a known radiosensitizer, with fractionated doses of [^177^Lu]Lu-DOTA-J591 176. The findings of the small (n = 15) study proved the feasibility of combining both chemo- and RIT in mCRPC. No dose limiting toxicities were observed with most patients displaying a dose-dependent decline in PSA.

Actively recruiting metals-based RIT clinical trials are currently minimal as described below, despite the number of FDA-approved naïve mAbs (e.g. trastuzumab, cetuximab) for a number of cancer indications. A phase I trial for patients with mCRPC using [^225^Ac]Ac-J591 (NCT03276572, projected completion Dec. 2021), and a phase II trial for patients with recurrent prostate cancer combining [^177^Lu]Lu-J591, ketoconazole and hydrocortisone with [^111^In]In-J591 as a placebo (NCT00859781, chelate not mentioned, projected completion Dec. 2022), are currently underway. Also, an active phase I study but currently not recruiting at the time of writing explores the benefit of [^177^Lu]Lu-DOTA-J591 for non-prostate metastatic solid tumors (NCT00967577, projected completion Dec. 2021). Additionally, a ^227^Th-labeled PSMA-specific mAb is in phase I studies, recruiting in Louisiana, Nebraska, New York, Finland and the United Kingdom, (NCT03724747, EudraCT: 2018-001490-24, projected completion date Sept. 2023). However, this study was suspended in Sweden.

Notably, a trial of ^177^Lu-labeled 5B1 targeting CA19-9 is currently active, but no longer recruiting in the United States and Germany (NCT03118349 projected completion date Dec. 2020). A first in human trial of [^227^Th]Th-anetumab, a mesothelin targeting radioimmunoconjugate, is actively recruiting patients with tumors known to express mesothelin (NCT03507452, EudraCT: 2017-004052-29, projected completion Nov, 2024) in the United States (Maryland and Texas), Finland, Sweden, the United Kingdom and the Netherlands. Finally, [^177^Lu]Lu-DTPA-Omburtamab, which targets B7-H3-expressing cells, is underway for an international Phase I/II RIT trial for pediatric and adolescent patients with recurrent or refractory medulloblastoma (NCT04167618, EudraCT: 2020-000670-22, projected completion Dec. 2024). The study is not yet recruiting at the time of writing, but trial countries include the United States, Denmark, Spain and the United Kingdom. [^177^Lu]Lu-DTPA-Omburtamab is also currently being investigated for treatment of leptomeningeal metastasis from solid tumors (NCT04315246, projected completion Dec. 2024) in the United States and the United Kingdom. Importantly, the trials with [^177^Lu]Lu-DTPA-Omburtamab take advantage of intracerebroventricular administration, which can mitigate the hematotoxicity seen in agents administered intravenously. A Phase I trial examining treatment of advanced solid tumors with [^90^Y]Y-FF-21101 targeting P-cadherin is underway. This study utilizes ^111^In-labeled FF-21101 to determine dosimetry (NCT02454010, estimated completion Dec. 2023). Finally, an international Phase I study targeting type I insulin-like growth factor receptor (IGF-1R) is exploring both single- and multi-dose escalation to determine the potential MTD and/or the recommended phase 2 dose of [^225^Ac]Ac-FPI-1434 for patients with advanced solid tumors (NCT3746431 estimated completion Dec. 2022).

## RIT moving forward

At the core of modern precision medicine is the idea that molecular alterations of cancer cells represent vulnerabilities that can be targeted with drugs. Despite sound mechanistic framework and strong bioplausibility, however, even patients with tumor bearing actionable who are treated with the corresponding targeted agent have modest response rates (<10%) that are unlikely to translate into improved overall survival 177. These findings reflect the dynamic, compensatory processes involved in oncogenesis, and present challenges to precision medicine as it is currently defined. In contrast, immune checkpoint inhibitors, the most exciting oncology agents that have demonstrated activity in diverse cancer types, do not target a specific tumor-associated antigen (TAA), but rather, enhance host immune response to recognize TAAs 178,179.

A notable question is where RIT might fit within the current paradigm of cancer therapy. While RIT as a modality has been around for several decades, it is important to emphasize the technological advances made in recent decades across the distinct disciplines required to engineer a RIT agent for preclinical and clinical testing. The diversity of antibody-based targeting molecules and ability to tune them to recognize targets of interest has improved dramatically. Commercial partnerships have allowed investigators to leverage external expertise, rather than having to build that infrastructure in-house, dramatically reducing costs to produce such bespoke molecules. The Department of Energy has expressed a commitment to ramp up production and quality of radioisotopes of clinical interest for therapy, including Lu-177, Ra-223, and Ac-225 to meet rising radionuclide demands for preclinical and clinical use. Chemists continue to improve on existing bifunctional chelates or discover new, more stable ones as highlighted earlier. The confluence of such advances has shortened the time interval between the identification of a molecule of interest and our ability to test the ligand-target pair *in vivo*, allowing investigators to more rapidly identify the most promising couple to translate to the clinic. Of course, none of these aforementioned advances matter were it not for institutional investments and/or commercial partnerships that can facilitate production of materials ready for human trials and sufficient funding to successfully execute them.

While the physical nature of certain radionuclides, especially those with high LET, may offer advantages over some molecular targeted therapies including lower likelihood of development of resistance 180, RIT combined with such drugs may prove beneficial. In fact, insights gained from clinical experience of other radiopharmaceutical therapies, such as those targeting PSMA in men with advanced prostate cancer, suggest that RIT combined with other agents may be needed to achieve durable tumor response rates and overall survival benefits 181. We have already commented on the natural synergy with DNA repair inhibitors, as well as tried and true radiosensitizers such as cisplatin and paclitaxel. Similarly, selection of patients with tumors bearing genomic lesions that result in enhanced sensitivity to DNA damage by ionizing radiation likely represent a cohort who may preferentially benefit from RIT. Furthermore, recent studies have also suggested that, like external beam radiotherapy (EBRT), RIT may enhance host anti-tumor immune response and synergize with immune checkpoint blockade 182,183. This is an area ripe for study and expands the concept of an *in situ* vaccine—the observation that ionizing radiation can induce an anti-tumor immune response—to many more sites than could be reasonably achieved by current external beam approaches 184. Because it offers distinct *in vivo* biodistribution compared to EBRT, RIT when combined with EBRT could allow intralesional dose escalation not possible with either modality alone, and may simultaneously allow additional sparing of organs at risk.

Deploying RIT and other radiopharmaceutical therapies will require close partnerships between preclinical and clinical development, not to mention addressing production and distribution challenges. Once an agent is brought to the clinic, collaborations between oncology (radiation, medical, and surgical), nuclear medicine, medical physicists, and radiation safety will be critical. In fact, rigorous training will be needed during residency and thereafter so this multidisciplinary team appropriately selects patients and can navigate the relevant acute and long term sequalae of both the disease and treatment. Such training appears to be more commonplace in programs outside the U.S. Furthermore, accurate dosimetry of RIT and other radiopharmaceutical therapies, that is, estimates of the absorbed dose to normal tissues and to tumor, can help inform treatment personalization and may affect clinical outcomes, though, this needs to be studied prospectively 185.

Recent enthusiasm for radiopharmaceutical therapies not only within nuclear medicine, but also in radiation and medical oncology, make evident that while prior iterations of RIT were met with some resistance to adoption and implementation, the current generation of agents has captured broader interest.

## Final Conclusions

The development of effective RIT agents for solid tumors requires a well-thought and streamlined strategy to achieve better outcomes for patients. Increased absorbed tumor dose coupled with minimal absorbed dose in off-target and healthy tissues is critical. Early stage preclinical development of RIT necessitates appropriate selection of radiometal, chelator, antibody format and a thorough *in vivo* investigation of its radiobiological effect. Despite the growing number of RIT agents developed in the preclinical pipeline, translation of the most promising agents has remained challenging. The need for expertise and infrastructure that incorporates nuclear medicine, medical oncology, radiation oncology, medical physics and appropriate radiation safety measures presents significant but not insurmountable barriers to translation. In summary, advances in the development of radiometal RIT agents may offer significant benefits in line with our goal of tailored, precision medicine for cancer treatment.

## Figures and Tables

**Table 1:** Radiometals for therapy with their half-life (t_1/2_), decay characteristics, path length in tissue and reported chelators

Radiometal	t_1/2_	Decay Properties (MeV)	Path Length	Reported Chelator(s)
**α-emitters**				
^223^Ra	11.4 d	5.8-7.53 (α) 17ˆ	46-68 μm 17	H_2_macropa 18
^225^Ac	9.9 d	5.8-8.4 (α) 19ˆˆ	47-85 μm^†^ 19	DOTA, H_2_macropa, Crown 20-26
^227^Th	18.7 d	6.14 (α) 27	^‡^	DOTA, Me-3,2-HOPO 28-30
^212^Pb^*^	10.6 h	6.05 (α)** 27		DOTA, TCMC 31,32
^212^Bi	60.6 min	6.05 (α, 36%) 27; 0.834 (β^-^, 64%) 27	51-92 μm 3	3p-C-DEPA, NETA, DOTA, CHX-A”-DTPA 29,30,33
^213^Bi	45.6 min	5.87 (α, 2.2%) 19,27; 0.492 (β^-^, 97.8%) 27	48-85 μm 3	3p-C-DEPA, NETA, DOTA, CHX-A”-DTPA 29,30,33
**β^-^-emitters**				
^177^Lu	6.7 d	0.497 (β^-^) 3	1.8 mm 3	DOTA, NETA, CHX-A”-DTPA 30,34-37
^90^Y	2.7 d	2.28 (β^-^) 3	11.3 mm 3	DOTA, NETA, CHX-A”-DTPA, DTPA 30,34,38-40
^67^Cu	2.6 d	0.395 (β^-^) 41	2.1 mm 3	DOTA, NOTA 42,43
^188^Re	17 h	2.12 (β^-^) 3,44	10.4 mm 3	Direct, MAG_2_-GABA, Trisuccin 45
^64^Cu	12.7 h	0.573 (β^-^, 38.4%) 41	0.95-1.4 mm 46	TETA, DOTA, NOTA; *p*-SCN-Bn-Oxo-DO3A, *p*-SCN-Bn-Oxo-PCTA 47
**Auger Electrons**		**AE Energy Released/Decay (keV)**		
^67^Ga	3.26 d	6.3 (AE) 48,49	0.002-2.1 μm 50	DFO, NOTA, DOTA, PCTA, *p*-NH_2_-Bn-Oxo-DO3A 30
^111^In	2.8 d	6.8 (AE) 48,49	2-500 nm 51	DOTA, CHX-A”-DTPA, H_4_octapa, NOTA, DTPA 52
^64^Cu	12.7 h	2 (AE, 41%) 53	126 nm 53	TETA, DOTA, NOTA; *p*-SCN-Bn-Oxo-DO3A, *p*-SCN-Bn-Oxo-PCTA 47

ˆ ^223^Ra yields four high-energy α-particles per disintegration. ˆˆ^ 225^Ac yields four α-particles per disintegration with energies ranging from 5.8 to 8.4 MeV. Three α-particles are emitted to decay to ^213^Bi, then one alpha particle is emitted from the two routes of decay to ^209^Bi. ^†^The range of α-emissions is defined by the α-emitting daughter isotopes of ^225^Ac. ^‡227^Th does not have a defined range of α-emissions due to successive α-emitting daughter isotopes.^29^ *^212^Pb is a β^-^ emitter but produces the daughter isotope ^212^Bi and is often used for targeted α therapy due to the short half-life of ^212^Bi. ** α energy emitted by ^212^Bi. Abbreviations: DOTA, 1,4,7,10-tetraazacyclododecane-1,4,7,10-tetraacetic acid; H_2_macropa, *N,N'*-bis[(6-carboxy-2-pyridil)methyl]-4,13-diaza-18-crown-6; Crown, 2,2',2'',2'''-(1,10-dioxa-4,7,13,16-tetraazacyclooctadecane-4,7,13,16-tetrayl)tetraacetic acid;Me-3,2-HOPO, 3-hydroxy-*N-*methyl-2-pyridinone; TCMC, 1,4,7,10-tetraza-1,4,7,10-tetra(2-carbamoylmethyl)cyclododecane; 3p-C-DEPA**,** 2-[(carboxymethyl)][5-(4-nitrophenyl-1-[4,7,10-tris(carboxymethyl)-1,4,7,10-tetraazacyclododecan-1-yl]pentan-2-yl)amino]acetic acid**;** NETA, ({4-[2-(Bis-carboxymethyl-amino_ethyl]-7-carboxymethyl-[1,4,7]triazonan-1-yl}; DTPA, diethylenetriamine pentaacetic acid; CHX-A”-DTPA, cyclohexane-1,2-diamine-pentaacetic acid; TETA, 1,4,8,11-tetraazacyclotetradecane-1,4,8,11-tetraacetic acid; NOTA, 1,4,7-triazacyclononane-1,4,7-triacetic acid; MAG_2_-GABA, S-ethoxyethyl mercapto-acetylglycylglycyl aminobutyrate; Trisuccin, N-[tris[2-[(N-hydroxyamino)carbonyl]ethyl]methyl]succinamic acid; *p*-SCN-Bn-Oxo-DO3A, 1-Oxa-4,7,10-triazacyclododecane-5-S-(4-isothiocyanatobenzyl)-4,7,10-triacetic acid; *p*-SCN-Bn-Oxo-PCTA, 3,6,9,15-tetraazabicyclo[9.3.1] pentadeca-1(15),11,13-triene-4-S-(4-isothiocyanatobenzyl)-3,6,9-triacetic acid; HBED, N,N'-bis (2-hydroxybenzyl) ethylenediamine-N,N'-diacetic acid; DFO, desferrioxamine; H_4_octapa, *N,N'*-bi(6-carboxy-2-pyridylmethyl)ethylenediamine-*N,N'*-diacetic acid).

**Table 2:** Comparison of radiometal RIT mAb formats developed for targeting EGFR

mAb	mAb Format	Radionuclide	Chelator	Dose Administered*	Study Highlights	Cancer cell line/xenograft
Panitumumab	Full-length	^212^Pb	TCMC	0.37-1.48 MBq	MS for 0.37 MBq and 0.74 MBq cohorts were 39 d and 58 d compared to 15 d for control untreated mice.	LS-174T i.p. xenografts 101
		^177^Lu	DOTA-AuNP^†^	1.5-4.5 MBq	A dose dependent decrease in *in vitro* clonogenic survival studies was observed.	MDA-MB-468 and MDA-MB-231 104
			DOTA	14.8 MBq	Tumor growth was inhibited up to 36 d p.i. compared to PBS and non-labeled control; no significant adverse events for body weight nor mortality noted.	UM-SCC-22B 105
			DOTA-MCP^‡^	6 MBq	A 6 MBq dosed activity in non-tumor bearing mice did not cause significant decreases in RBC, WBC or platelets, no increase in serum ALT and only a small increase in Cr. [^177^Lu]Lu-MCP-panitumumab administered mice exhibited significantly decreased tumor volumes at 33 d p.i. compared to control.	PANC-1 107
	F(ab)'_2_	^212^Pb	TCMC	0.37-3.7 MBq i.p. 0.185-1.85 MBq	1.11 MBq (i.p.) and 0.74 MBq (i.v.) were selected as effective therapeutic doses with MS of 289 d and 46 d, respectively. Although benefit of i.p. was noted, i.v. administration was chosen for co-administration with gemcitabine (MS: 208 d) or paclitaxel (MS: 239 d).	LS-174T i.p. 119
		^64^Cu	NOTA	1.85-9.25 MBq	3.7 MBq administered every two weeks was selected. No generalized toxicity of the tracer was noted.	OCIP23 pancreatic PDX and PANC-1 120
Cetuximab	Full-length	^177^Lu	DOTA	14.8 MBq	A significant tumor growth delay was observed up to 30 d p.i., but tumors grew significantly larger (>1500 mm^3^ 35 d p.i.) compared to [^177^Lu]Lu-DOTA-panitumumab; no significant adverse events for body weight nor mortality noted.	UM-SCC-22B 105
			PCTA	12.95 MBq	A significant difference in tumor volume 16 d p.i. was observed compared to saline or non-labeled cetuximab controls.	TE-8 109
				12.95 MBq	A 55% reduction in tumor volume after treatment was observed. There was a significant decrease in final tumor volume 30 d p.i. compared to saline and non-labeled cetuximab controls.	SNU-1066 111
		^188^Re	N/A	22.2-59.2 MBq	MTD was determined to be 37 MBq. Treatment studies were conducted with 29.6 and 22.2 MBq with MS of 62.5 and 61.75 d (control MS: 36.75 d).	NCI-H292 108
		^64^Cu	PCTA	11.1-74 MBq	MTD: 22.2 MBq. Survival of mice was at 40% when treated with adjuvant [^64^Cu]Cu-PCTA-cetuximab at 83 d with no detectable lesions.	x-PA-1-DC orthotopic xenograft 110
		^212^Pb	TCMB	0.37-1.48 MBq	0.37 MBq was chosen as the effective therapeutic dose due to lack of toxicity and a MS that lasted beyond 294 d.	LS-174T i.p. xenografts 113
	F(ab)'_2_	^177^Lu	DOTAGA	2-8 MBq	Colorectal tumor growth was inhibited for mice administered 4 and 8 MBq compared to 2 MBq and control. Acute weight loss was observed at the 4 MBq dose 20 d p.i. and mice recovered by 23 d p.i.	A431 112

^†^AuNP: Gold nanoparticles used for radiosensitization; ^‡^MCP: Metal chelating polymers; *All activities administered i.v. unless otherwise noted. Abbreviations: TCMC, 1,4,7,10-tetraza-1,4,7,10-tetra(2-carbamoylmethyl)cyclododecane; MS, median survival; RBC, red blood cells; WBC, white blood cells; ALT, alanine aminotransferase; Cr, creatinine; MTD, maximum tolerated dose; PCTA, 3,6,9,15-tetraazabicyclo[9.3.1]pentadeca-1(15),11,13-triene-3,6,9-triacetic acid.

**Table 3:** Innate characteristics of common antibody platforms used in radiopharmaceutical development categorized as either engineered or enzymatically produced

		Enzymatic		Engineered				
	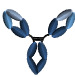	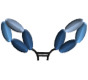	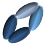	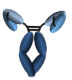	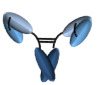	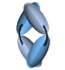	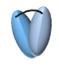	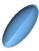
Format	Intact	F(ab')_2_	Fab	scFv-Fc	Minibody	Diabody	scFv	Nanobody
MW (kDa)	150	110	55	105	80	55	28	12-15
Valency	Divalent	Bivalent	Monovalent	Bivalent	Bivalent	Bivalent	Monovalent	Monovalent
Serum Half-life	1-3 weeks 125	8-10 h 125	12-20 h 125	8-80 h 126 (Terminal)	5-10 h 125	5-6 h 125	2-4 h 125	0.5-1 h 125
Clearance Route	Liver 127	Liver, Kidney 123,127	Kidney 127	Liver	Liver 127	Kidney 127	Kidney 127	Kidney 124

**Table 4:** Comparison of radiometal RIT mAb formats developed for HER2 and CEA

mAb Format	mAb	Radionuclide	Chelator	Dose Administered	Study Highlights	Cancer cell line/xenograft
**HER2**						
Full-mAb	Trastuzumab	^90^Y	IB4M-DTPA	1.48-2.96 MBq	Variations in RIT dose and combined therapy with taxol. Tumor regression was observed over 35 d post treatment compared to controls. No toxicity studies were examined.	MCF-7 140
		^177^Lu	SPIONs	0.1 MBq	Highest uptake was observed in the liver and spleen. Neither toxicity studies, nor therapeutic efficacy for tumors were examined.	SKOV-3 141
			DOTA	11.1-55.5 MBq	Single and fractionated cycles were examined at various activities for small (palpable-30 mm^3^) and medium (100-400 mm^3^) tumors. Complete response was observed in the fractionated triple cycle of 55.5 MBq in medium sized tumors.	BT474^‡^ 71
		^213^Bi	CHX-A”-DTPA	1.85 MBq	MS of [^213^Bi]Bi-CHX-A”-DTPA-trastuzumab combined with carboplatin 24 h post RIT (87 d) was longer than control (17 d). A combination of [^213^Bi]Bi-CHX-A”-DTPA-trastuzumab with three doses of carboplatin starting 24 h post RIT increased MS two-fold (186 d) compared to control (23 d).	LS-174T i.p. 142
		^212^Pb	TCMC	0.37 MBq	No significant difference in MS (based on timing of carboplatin).	LS-174T i.p. 142
				0.37-1.48 MBq	Comparison of internalizing ^212^Pb-labeled trastuzumab to non-internalizing [^212^Pb]Pb-TCMC-35A7. The MS for [^212^Pb]Pb-TCMC-trastuzumab was not reached after 130 d. A final absorbed dose of 27.6 Gy was observed.	A431 i.p. xenografts 129
		^227^Th	DOTA	0.2-0.6 MBq/kg	Significant increase in MS for 0.4 MBq/kg (63 ± 3 d) and 0.6 MBq/kg (96 ± 3 d) compared to saline control (42 ± 13 d).	SKBR-3 143
Fab	Bispecific Trastuzumab	^111^In/^177^Lu	DTPA/DOTA	3.7-18.5 MBq	RBCs, Hb and HCT were significantly lower for mice receiving 18.5 MBq, with no significant difference for serum ALT and Cr at any dose; 11.1 MBq chosen for RIT studies. Tumor growth was inhibited 1.6-fold.	SKOV-3; MDA-MB-231 139
Diabody	C6.5K-A	^90^Y	CHX-A”-DTPA	1.85-18.5 MBq	7.4 MBq for MDA-361/DYT2 and 11.1 MBq for SKOV-3 exhibited a nine- and three-day delay in doubling time.	SKOV-3; MDA-361/DYT2 138
Nanobody	2Rs15d	^177^Lu	*p-*SCN-Bn-DOTA; DOTA-NHS-ester; CHX-A”-DTPA; 1B4M-DTPA	20 MBq	Expected toxicity is noted for the kidney (195 %IA/g). No other observations on tumor response or toxicity studies noted.	LS174-T; SKOV-3; MDA-MB-435D 36
			DTPA	21.5 MBq	7/8 mice reached event free survival up to 125 d p.i. All controls euthanized by 85 d p.i.	SKOV-3 37
		^225^Ac	DOTA	0.0293 MBq	Co-administration with Gelofusin significantly decreased renal accumulation by three-fold. Therapeutic efficacy and tumor growth inhibition were not examined.	SKOV-3; MDA-MB-231 20
				0.0659 MBq	MS SKOV-3.IP1: ^225^Ac]Ac-2Rs15d + trastuzumab: 29.5 d; [^225^Ac]Ac-2Rs15d: 23 d; Trastuzumab: 19 d; Control: 17 d. MS MDA-MB-231Br: ^225^Ac]Ac-2Rs15d + trastuzumab: 30 d; [^225^Ac]Ac-2Rs15d: 34 d; Trastuzumab: 24.5 d; Control: 22 d.	Intracranial tumors of SKOV-3.IP1 & MDA-MB-231Br 136
**CEA**						
Full-mAb	cT84.66	^90^Y	DOTA	0.74-3.7 MBq	Variation of [^90^Y]Y-DOTA-cT84.66 alone or in combination with taxol or cold trastuzumab,	MCF-7 140
	35A7	^177^Lu	DOTA-Tz	40 MBq/250 μL	Pretargeted RIT assessment of various Tz-PEG_n_ linkers to optimize tumor uptake and clearance profiles.	Orthotopic peritoneal carcinomatosis 96
		^212^Pb	TCMC	0.37-1.48 MBq	Comparison of internalizing ^212^Pb-labeled trastuzumab to non-internalizing [^212^Pb]Pb-TCMC-35A7. A MS = 94 d for [^212^Pb]Pb-TCMC-35A7 was observed with a final absorbed dose of 35.5 Gy.	A431 i.p. xenografts 129

^‡^ An IgG-scFv bispecific format was utilized with the IgG sequence of Trastuzumab. Abbreviations: IB4M, 2-(*p*-isothiocyanatobenzyl)-6-methyl-diethylenetriamine-*N*,*N*,*N*´,*N*´´,*N*´´-pentaacetic acid; SPIONs, Super Paramagnetic Iron Oxide Nanoparticles; MS, Median Survival; RBC, red blood cells; Hb, hemoglobin; HCT, hematocrit; ALT, alanine aminotransferase; Cr, creatinine; %IA/g, percent injected activity per gram.
